# Prevalence and Risk Factors of Low Back Pain Among Healthcare Workers in Major Hospitals in Al Madinah, Saudi Arabia: A Cross-Sectional Study

**DOI:** 10.7759/cureus.100670

**Published:** 2026-01-03

**Authors:** Ahmed Almuhammady, Osama Alamri, Abdullah Alsehli, Turki Alharbi, Abdullah Alharbi, Muath Alferadi, Jaser Aljohani, Mohammed Abdullah, Abdulaziz Aljohani, Omar Afghani, Abdulilah Hamdan, Ahmed Abdelaal

**Affiliations:** 1 College of Medicine, Taibah University, Madinah, SAU; 2 Orthopaedic Surgery, King Salman Bin Abdulaziz Medical City, Madinah, SAU; 3 Orthopaedic Surgery, Taibah University, Madinah, SAU

**Keywords:** healthcare workers, low back pain, musculoskeletal disorders, occupational risk factors, prevalence

## Abstract

Background: Low back pain (LBP) is a significant occupational health burden and the most common musculoskeletal condition among healthcare professionals worldwide. Data on its prevalence and contributing factors in major hospitals in Al Madinah remain limited.

Methods: A cross-sectional survey was conducted among 382 healthcare professionals from major hospitals in Al Madinah using a standardized, self-administered questionnaire. Descriptive statistics (frequencies and percentages), chi-square and Fisher's exact tests, and multivariable binary logistic regression were performed using IBM SPSS Statistics for Windows, Version 26.0 (IBM Corp., Armonk, NY, USA). Independent predictors of LBP were reported as adjusted odds ratios (OR) with 95% confidence intervals (CI), and statistical significance was set at p < 0.05.

Results: The lifetime prevalence of LBP was 85.9%, with 73.6% reporting symptoms in the past year and 43.7% experiencing current pain. Independent predictors of LBP included a family history of LBP (OR = 2.19, p = 0.021), working ≤8 hours/day (OR = 2.18, p = 0.017), standing ≤5 hours/day (OR = 0.48, p = 0.023), and smoking (OR = 0.54, p = 0.049). After adjustment, work-related physical tasks and stress level were not significant predictors.

Conclusion: LBP is highly prevalent among healthcare workers in Al Madinah. Familial predisposition and specific work-hour patterns emerged as key determinants. These findings underscore the need for targeted ergonomic and organizational interventions and support future longitudinal studies to clarify causal pathways.

## Introduction

Pain is an uncomfortable emotional and sensory experience that arises in the brain in response to stimuli from internal regions of the body. It acts as a defense mechanism, helping an individual protect an injured body part from further harm. Low back pain (LBP), also known as lumbago or lumbosacral pain, typically occurs above the gluteal folds and below the twelfth rib [1]. LBP was once considered primarily a Western health issue, but over the past decade, research has increasingly shown that it is also a significant problem in low- and middle-income countries [2-5]. Globally, the prevalence of LBP ranges from 15% to 45% of the population [3]. In Saudi Arabia, studies report a prevalence of 18.8% in the general population [4]. Among healthcare professionals, the prevalence ranges from 33% to 68%, with female workers experiencing slightly higher rates than males [5,6]. LBP is among the most common conditions requiring medical attention. Healthcare workplaces, in particular, have higher rates of occupational injuries and musculoskeletal disorders, with LBP representing one of the most debilitating concerns [7]. Studies in Saudi Arabia indicate that the prevalence of LBP among healthcare workers varies regionally, with 80% reported in Riyadh and 61.7% in Jeddah [1,8]. In the United States, back pain is the leading cause of discomfort, with an annual point prevalence averaging 30% and a range of 15% to 45%. Among active healthcare professionals in Saudi Arabia, LBP may negatively impact clinical productivity. It is the most common cause of activity limitation in individuals under 45 and the second most frequent reason for doctor visits [9]. According to the World Health Organization (WHO), musculoskeletal disorders, including LBP, are leading contributors to disability and limitations in daily functioning and employment [10]. Approximately 2% of US workers receive compensation for back injuries each year [9]. Moreover, LBP is the second most common reason for missed work, resulting in greater productivity loss than almost any other condition [11,12]. Numerous studies have identified a variety of risk factors for LBP in the general population. These include older age, substance use, gender, family history, obesity, poor posture, prior back injury, and smoking. Occupational factors, such as manual lifting and repositioning of patients, insufficient breaks, and work in surgical departments, have also been linked to higher LBP prevalence [2,13-16]. Social and psychological factors further contribute to the risk. Although studies have explored LBP in Saudi Arabia, gaps remain, particularly regarding healthcare workers in the Al Madinah region and the identification of associated risk factors.

Therefore, this study aimed to determine the prevalence of LBP and identify its associated risk factors among healthcare workers at major hospitals in Al Madinah, Saudi Arabia.

## Materials and methods

Study design

This was a descriptive cross-sectional study aimed at determining the prevalence of LBP and its associated risk factors among healthcare workers (HCWs) in major hospitals in Al Madinah. Data were collected between August and October 2025 using a validated, self-administered online questionnaire adapted from a study by Gweha et al. [17], which investigated the prevalence and associated factors of LBP among HCWs in Cameroon. The questionnaire was freely available for academic use without license restrictions. It was distributed online via Google Forms (Google, Mountain View, CA, USA) to HCWs at selected major hospitals in Al Madinah. The questionnaire included three sections: (1) sociodemographic and lifestyle characteristics (age, gender, marital status, tobacco use, chronic diseases, family history of LBP, body mass index (BMI), stress level, and physical activity); (2) occupational factors (job category, department, years of work, working hours, standing and seated hours, monthly on-call shifts, patient lifting, bending, and transferring/ambulating patients); and (3) health and LBP-related history (lifetime, annual, and current LBP). A non-probability convenience sampling technique was used.

Sample size calculation 

The Raosoft sample size calculator (Raosoft Inc., Seattle, WA, USA) was used to determine the required sample size. The total population of HCWs in the selected hospitals was 15,819 (physicians 3,410; nurses 6,457; pharmacy professionals 237; other auxiliary medical staff 5,715), according to the Saudi Ministry of Health Annual Statistical Book 2023. Using a 5% margin of error, 95% confidence level, and 50% response distribution, the recommended sample size was 376. To ensure adequate representation and improve robustness, 382 participants were included. 

Inclusion and exclusion criteria

All HCWs (physicians, nurses, pharmacy professionals, medical imaging staff, laboratory staff, and physiotherapists) who had been working in the selected hospitals for at least 12 months were included. The hospitals included King Fahad General Hospital, Madinah General Hospital, Maternity and Child Hospital, and Ohud Hospital. HCWs with back surgery or trauma in the previous 12 weeks, pregnancy or childbirth within the last three months, or medical conditions known to cause LBP (e.g., infection, tumor or metastasis, vertebral fracture, spinal stenosis, visceral disease, inflammatory disorders, spinal deformity, or cauda equina syndrome) were excluded.

Data collection

Data were collected using the online self-administered questionnaire via Google Forms. The questionnaire link was distributed by the research team through official institutional communication channels and professional messaging groups. Participation was voluntary, and data were collected over three months. Only fully completed questionnaires were included in the final analysis; incomplete responses were automatically excluded.

Data analysis

Statistical analysis was performed using IBM SPSS Statistics for Windows, Version 26.0 (IBM Corp., Armonk, NY, USA). Descriptive statistics were used to summarize the data. Categorical variables were presented as frequencies and percentages. Associations between categorical variables were analyzed using the chi-square test or Fisher’s exact test when assumptions were not met. Multivariable binary logistic regression analysis was performed to identify independent predictors of LBP. Results were reported as adjusted odds ratios (ORs) with 95% confidence intervals (CIs) using Jamovi Software version 2.7.6. Statistical significance was set at p < 0.05.

Ethical consideration

Ethical approval for this study was obtained from the Scientific Research Ethics Committee, College of Medicine, Taibah University, Saudi Arabia (Study ID: STU-25-020). All participants were informed about the study’s aims and provided electronic informed consent before participation. Participation was voluntary, and respondents could withdraw at any time without consequences. No identifying information was collected, and all data were anonymized and stored securely to ensure confidentiality and compliance with ethical standards.

## Results

Our study included 382 participants, the majority of whom were aged 20-39 years. Most were male (62%) and married (60.5%), with nearly half (48.4%) having a normal body weight. Tobacco use was reported by 124 participants, while 258 were non-smokers. Approximately 23% had chronic diseases, and 40.6% reported a family history of LBP. Regarding stress and physical activity, most participants (50.8%) reported a moderate perceived stress level, followed by a high stress level, and 49.7% had a moderate level of physical activity (Table 1).

**Table 1:** Sociodemographic characteristics of healthcare workers at major hospitals in Al Madinah (N = 382)

Factor	Category	Number	Percent
Age (years)	20-29	139	36.4
	30-39	138	36.1
	40 years and older	105	27.5
Gender	Female	145	38.0
	Male	237	62.0
Marital status	Married	231	60.5
	Single	151	39.5
Smoking tobacco	No	258	67.5
	Yes	124	32.5
Chronic diseases	No	294	77.0
	Yes	88	23.0
BMI (kg/m^2^)	Underweight (<18.5)	17	4.5
	Normal weight (18.5-24.9)	185	48.4
	Overweight (25-29.9)	140	36.6
	Obese (>30)	40	10.5
Do you have a family history of low back pain?	No	227	59.4
	Yes	155	40.6
How would you rate your stress level over the last month?	Low	54	14.1
	Moderate	194	50.8
	High	134	35.1
How physically active are you?	Low	125	32.7
	Moderate	190	49.7
	High	67	17.5

Physicians were the most represented professional group, with 176 participants (46.1%), followed by nurses with 140 participants (36.6%). The medicine and specialties department accounted for the largest proportion of workers (113; 29.6%), followed closely by the surgery department (110; 28.8%). Most participants had 1-11 years of work experience (64.7%). Regarding work patterns, slightly over half of the participants (n = 196; 51.3%) worked ≤8 hours per day, 218 (57.1%) stood for more than 5 hours daily, and 266 (69.6%) were seated for more than 5 hours per day. Among respondents, 153 (40.1%) reported lifting heavy objects, 269 (70.4%) reported frequent bending, and 171 (44.8%) reported transferring or ambulating patients (Table 2).

**Table 2:** Work-related characteristics of healthcare workers at major hospitals in Al Madinah (N = 382) *Other: laboratory professional, medical imaging professional, physiotherapist. **Other: maternity, ICU, laboratory, radiology, theater, emergencies.

Factor	Category	Number	Percent
Job category	Nursing	140	36.6
	Pharmacy professional	29	7.6
	Physician	176	46.1
	Other*	37	9.7
Department	Medicine and specialties	113	29.6
	Pediatric	42	11
	Pharmacy	27	7.1
	Surgery	110	28.8
	Other**	90	23.6
How many years have you been working?	1-11 years	247	64.7
	≥12 years	135	35.3
How many hours do you work daily on average?	≤8 hours	196	51.3
	>8 hours	186	48.7
How many hours do you spend standing at work daily?	≤5 hours	164	42.9
	>5 hours	218	57.1
How many hours do you spend sitting at work daily?	≤5 hours	116	30.4
	>5 hours	266	69.6
How many monthly calls do you typically handle?	≤4	235	61.5
	>4	147	38.5
Do your job duties involve lifting heavy objects?	No	229	59.9
	Yes	153	40.1
Do your job duties involve bending to work?	No	113	29.6
	Yes	269	70.4
Do your job duties involve transferring/ambulating patients?	No	211	55.2
	Yes	171	44.8

Table 3 summarizes the prevalence of LBP among healthcare workers. Of the 382 participants, 328 (85.9%) reported experiencing LBP at some point in their lifetime, 281 (73.6%) reported LBP within the past 12 months, and 167 (43.7%) reported current LBP at the time of the study.

**Table 3:** Occurrence of lifetime, annual, and current low back pain among healthcare workers at major hospitals in Al Madinah (N = 382)

Factor	Category	Number	Percent
Have you ever experienced low back pain in your lifetime?	No	54	14.1
	Yes	328	85.9
Have you experienced low back pain in the past 12 months?	No	101	26.4
	Yes	281	73.6
Are you currently experiencing low back pain?	No	215	56.3
	Yes	167	43.7

Table 4 presents the sociodemographic factors associated with LBP. Smoking status was significantly associated with LBP, with non-smokers showing a higher prevalence (n = 228; 88.4%) compared to smokers (p = 0.042). A family history of LBP was also significantly associated with LBP, with 140 participants (90.3%) affected compared to those without a family history (p = 0.039). Stress level was another significant factor (p = 0.042), with participants reporting moderate stress (n = 159; 82%) and high stress (n = 123; 91.8%), showing the highest prevalence of LBP compared with those reporting low stress.

**Table 4:** Sociodemographic factors associated with lifetime low back pain among healthcare workers at major hospitals in Al Madinah *Chi-square test. **Fisher’s test. Statistical significance was set at p < 0.05.

Factor	Category	Low back pain lifetime, N (%)		p-value	χ²
		No	Yes		
Age (years)	20-29	21 (15.1)	118 (84.9)	0.914*	0.179
	30-39	19 (13.8)	119 (86.2)		
	40 years and older	14 (13.3)	91 (86.7)		
Gender	Female	18 (12.4)	127 (87.6)	0.450*	0.571
	Male	36 (15.2)	201 (84.8)		
Marital status	Married	31 (13.4)	200 (86.6)	0.619*	0.247
	Single	23 (15.2)	128 (84.8)		
Smoking tobacco	No	30 (11.6)	228 (88.4)	0.042*	4.12
	Yes	24 (19.4)	100 (80.6)		
Chronic diseases	No	39 (13.3)	255 (86.7)	0.372*	0.797
	Yes	15 (17)	73 (83)		
BMI (kg/m^2^)	Underweight (<18.5)	3 (17.6)	14 (82.4)	0.450**	2.629
	Normal weight (18.5-24.9)	31 (16.8)	154 (83.2)		
	Overweight (25-29.9)	16 (11.4)	124 (88.6)		
	Obese (>30)	4 (10)	36 (90)		
Do you have a family history of low back pain?	No	39 (17.2)	188 (82.8)	0.039*	4.272
	Yes	15 (9.7)	140 (90.3)		
How would you rate your stress level over the last month?	Low	8 (14.8)	46 (85.2)	0.042*	6.336
	Moderate	35 (18)	159 (82)		
	High	11 (8.2)	123 (91.8)		
How physically active are you?	Low	13 (10.4)	112 (89.6)	0.073*	5.228
	Moderate	26 (13.7)	164 (86.3)		
	High	15 (22.4)	52 (77.6)		

Table 5 summarizes the association between the work-related factors and the LBP. No significant differences were observed in work-related factors or in the prevalence of low back pain.

**Table 5:** Work-related factors associated with lifetime low back pain among healthcare workers at major hospitals in Al Madinah *Fisher’s test. **Chi-square test. Statistical significance was set at p < 0.05.

Factor	Category	Low back pain lifetime, N (%)		p-value	χ²
		No	Yes		
Job category	Nursing	14 (10)	126 (90)	0.144*	5.351
	Pharmacy professional	4 (13.8)	25 (86.2)		
	Physician	27 (15.3)	149 (84.7)		
	Other	9 (24.3)	28 (75.7)		
Department	Medicine and specialties	16 (14.2)	97 (85.8)	0.609*	2.894
	Pediatric	3 (7.1)	39 (92.9)		
	Pharmacy	3 (11.1)	24 (88.9)		
	Surgery	16 (14.5)	94 (85.5)		
	Other	16 (17.8)	74 (82.2)		
How many years have you been working?	1-11 years	31 (12.6)	216 (87.4)	0.229**	1.448
	≥12 years	23 (17)	112 (83)		
How many hours do you work daily on average?	≤8 hours	22 (11.2)	174 (88.8)	0.094**	2.812
	>8 hours	32 (17.2)	154 (82.8)		
How many hours do you spend standing at work daily?	≤5 hours	29 (17.7)	135 (82.3)	0.084**	2.978
	>5 hours	25 (11.5)	193 (88.5)		
How many hours do you spend sitting at work daily?	≤5 hours	34 (12.8)	232 (87.2)	0.250**	1.323
	>5 hours	20 (17.2)	96 (82.8)		
How many monthly calls do you typically handle?	≤4	29 (12.3)	206 (87.7)	0.203**	1.622
	>4	25 (17)	122 (83)		
Do your job duties involve lifting heavy objects?	No	32 (14)	197 (86)	0.911**	0.012
	Yes	22 (14.4)	131 (85.6)		
Do your job duties involve bending to work?	No	22 (19.5)	91 (80.5)	0.052**	3.76
	Yes	32 (11.9)	237 (88.1)		
Do your job duties involve transferring/ambulating patients?	No	25 (11.8)	186 (88.2)	0.154**	2.033
	Yes	29 (17)	142 (83)		

In the multivariate regression model, several factors remained significantly associated with LBP. Smoking was associated with lower odds of LBP (OR = 0.540, 95% CI: 0.292-0.998, p = 0.049). A family history of LBP significantly increased the likelihood of experiencing LBP (OR = 2.190, 95% CI: 1.127-4.256, p = 0.021). Working ≤8 hours per day was also associated with higher odds of LBP (OR = 2.180, 95% CI: 1.151-4.130, p = 0.017). Additionally, standing for ≤5 hours per day was significantly associated with LBP (OR = 0.480, 95% CI: 0.255-0.903, p = 0.023).

**Table 6:** Multivariate regression analysis of predictors of reported low back pain Statistical significance was set at p < 0.05.

Factors	Having low back pain, OR (95%CI)	p-value
Smoking tobacco (No)		
Yes	0.540 (0.292 - 0.998)	0.049
Do you have a family history of low back pain (No)		
Yes	2.190 (1.127 - 4.256)	0.021
How many years have you been working (1-11 years)		
≥12 years	0.701 (0.377 - 1.306)	0.263
How many hours do you work daily on average (>8 hours)		
≤8 hours	2.180 (1.151 - 4.130)	0.017
How many hours do you spend standing at work daily (>5 hours)		
≤5 hours	0.480 (0.255-0.903)	0.023
How would you rate your stress level over the last month (low)		
Moderate	0.720 (0.304-1.708)	0.456
High	1.904 (0.699-5.185)	0.208
Do your job duties involve transferring/ambulating patients (No)		
Yes	0.617 (0.336-1.135)	0.121

## Discussion

This study demonstrated a high prevalence of LBP among HCWs in major hospitals in Al Madinah, with family history, working hours, standing duration, and smoking status identified as significant associated factors. Several previous studies have highlighted LBP as the most prevalent musculoskeletal injury among HCWs [18], with documented effects on healthcare system performance and worker productivity [19]. Identifying factors associated with LBP is crucial for designing effective interventions and improving working conditions for HCWs [1].

The prevalence of LBP in our study was notably high, with 85.9% reporting lifetime LBP, 73.6% reporting LBP in the past 12 months, and 43.7% reporting current LBP. These rates are higher than those reported in international systematic reviews, where the pooled lifetime prevalence among HCWs was 54% [20]. Our prevalence results also exceed those observed in the general population in Turkey, which reported a lifetime risk of 44.1% and a 12-month recall rate of 34% [21]. Studies in other countries likewise report lower prevalence rates among HCWs, including 46% in Nigeria [22], 54.7% in Japan [23], 51% in Tunisia [24], and 30% in Ireland [25]. However, our findings are more comparable to studies from similar regional and socioeconomic contexts. For example, Alnaami et al. [7] documented a 12-month prevalence of 73.9% among HCWs in southwestern Saudi Arabia, and an Ethiopian study reported an 80% prevalence among nurses [26].

Family history emerged as a significant predictor of LBP in our analysis (p = 0.021), with 90.3% of participants with a family history reporting LBP versus 82.8% without such a history. This finding supports the growing body of evidence recognizing the genetic and familial contributions to LBP. For example, the Norwegian HUNT study found that spinal pain prognosis is significantly influenced by familial factors [27]. Twin studies further demonstrate that genetic heritability contributes substantially to both LBP risk and lumbar disc degeneration, with approximately 34% of genetic effects shared across both conditions [28].

In our multivariate analysis, smoking showed a statistically significant association with LBP (OR = 0.540, p = 0.049), but the direction of the association suggested lower odds of LBP among smokers. This contradicts well-established evidence linking smoking with musculoskeletal degeneration and is likely explained by residual confounding, differences in job roles, or reporting bias rather than a true protective effect.

A notable finding was the higher prevalence of LBP among workers who reported working ≤8 hours per day (p = 0.017), compared to those working longer hours. This pattern is likely driven by reverse causation, where workers with LBP may self-limit their work hours or be assigned shorter shifts due to existing symptoms. Other studies, such as Guo et al.’s analysis of the 2010 National Health Interview Survey, demonstrated that longer work hours were associated with increased LBP risk, particularly among young men (>60 hours/week) and women working 41-45 hours/week [29]. This suggests that work intensity, rather than duration alone, may be a more relevant factor. Physical activity was not associated with LBP in our study, similar to findings from Mansoura Hospital in Egypt, which reported no significant relationship between physical activity levels and LBP risk [30]. Standing for more than 5 hours per day was found to increase the risk of LBP (p = 0.023). This aligns with previous evidence indicating that prolonged standing is a key occupational hazard contributing to musculoskeletal disorders. For example, Ayane et al. reported that those exposed to prolonged standing had five times the odds of developing LBP [31].

Other measures of workload exposure, such as total hours worked per week, shift type, and duration of rest breaks, may serve as more refined predictors of LBP risk. However, due to the cross-sectional nature of our design, these temporal relationships cannot be fully assessed. Prospective studies are needed to better understand how different aspects of work schedules and demands influence LBP over time.

Implications

These findings highlight the need for targeted ergonomic and organizational interventions aimed at reducing prolonged standing and optimizing work schedules among HCWs. Implementing ergonomic training, redesigning workflow to distribute physical demands, and ensuring adequate rest breaks may help mitigate LBP risk. Early identification of high-risk individuals, particularly those with a positive family history, may also support timely preventive strategies.

Limitations

This study has several limitations. First, the cross-sectional design restricts causal inference and allows for the possibility of reverse causation in some observed associations. Second, all data were self-reported, introducing the potential for recall bias and limiting the precision of exposure and outcome measurements. Third, the study was conducted in a single geographic region, which may limit the generalizability of the findings to HCWs in different settings with varying work environments, cultural contexts, or healthcare system structures.

## Conclusions

Our study identified a concerning situation regarding LBP among healthcare staff in Al Madinah’s major hospitals, with a notably high prevalence that signals an important occupational health issue. The strong associations with family history and specific work-hour patterns emphasize that LBP in clinical settings has multiple, interconnected causes. Our findings point to a complex mix of inherited factors, physical demands, and workplace management challenges. Future research using prospective longitudinal designs is recommended to clarify causal pathways and support the development of more effective prevention strategies.
